# CIRRHOSIS-ASSOCIATED SARCOPENIA: INTEGRATING IMAGING, FUNCTION, AND METABOLISM FOR CLINICAL DECISION-MAKING

**DOI:** 10.1590/S0004-2803.24612026-017

**Published:** 2026-08-14

**Authors:** Aloma Conceição Magno CAMPECHE-LEÃO, Monalisa Reis ARRUDA, Ana Clara Vital BATISTA, Lais Spindola GARCEZ, Rosangela Passos de JESUS, Helma Pinchemel COTRIM, Lourianne Nascimento CAVALCANTE

**Affiliations:** ¹ Universidade Federal da Bahia, Programa de Pós-Graduação em Medicina e Saúde (PPGMS), Salvador, BA, Brasil.; 2 Universidade Federal da Bahia, Programa de Pós Graduação em Alimentos, Nutrição e Saúde, Salvador, BA, Brasil.

**Keywords:** Cirrhosis, frailty, muscle mass, nutrition, Cirrose, sarcopenia, fragilidade, massa muscular, nutrição

## Abstract

**Background:**

Sarcopenia is a highly prevalent and clinically impactful complication of cirrhosis, affecting 30-70% of patients depending on diagnostic criteria and disease stage. It is strongly associated with increased mortality, hepatic decompensation, infections, waitlist dropout, and inferior posttransplant outcomes. Despite growing recognition, the field remains limited by heterogeneous definitions, lack of standardized cutoffs, and inconsistent integration of functional and structural assessments.

**Objective and Methods:**

This narrative review synthesizes mechanistic pathways linking cirrhosis to muscle wasting, including hyperammonemia, anabolic resistance, systemic inflammation, hormonal dysregulation, and mitochondrial dysfunction, and critically evaluates current diagnostic tools such as CT morphometry, DEXA, BIA, and functional performance tests. Emerging biomarkers (myostatin, irisin, metabolomic signatures) and artificial intelligence-based imaging approaches are discussed as promising but still immature tools requiring validation.

**Conclusion:**

Therapeutic strategies remain constrained by low-quality evidence, with nutritional optimization, resistance training, and management of hepatic complications forming the cornerstone of care. Novel anabolic and anticatabolic therapies show potential but lack robust clinical trial data. Standardization of diagnostic criteria, integration of multimodal assessments, and development of targeted interventions represent urgent priorities to improve outcomes in cirrhosis-associated sarcopenia.

## HIGHLIGHTS

• Sarcopenia affects up to 70% of patients with cirrhosis and independently predicts mortality and decompensation.

• Muscle strength is a stronger prognostic marker than muscle mass alone.

• CT-based morphometry is the most validated structural tool, but multimodal assessment is essential.

• Nutritional optimization and structured exercise are the most consistently beneficial interventions.

• Emerging biomarkers show promise but lack validation for clinical use.

• Harmonized diagnostic criteria and standardized endpoints are urgently needed.

## Resumo

**Contexto:**

A sarcopenia associada à cirrose é uma complicação frequente e clinicamente relevante, com prevalência estimada entre 30% e 70%, estando associada a maior mortalidade, descompensação hepática, infecções e piores desfechos pós-transplante. Apesar de sua importância prognóstica, persistem limitações relacionadas à heterogeneidade conceitual, ausência de pontos de corte padronizados e integração insuficiente entre avaliações estruturais e funcionais.

**Objetivo:**

Revisar criticamente os mecanismos fisiopatológicos, métodos diagnósticos e estratégias terapêuticas da sarcopenia na cirrose, integrando evidências atuais e perspectivas emergentes.

**Métodos:**

Revisão narrativa da literatura baseada em estudos observacionais, ensaios clínicos, diretrizes internacionais e revisões sistemáticas publicadas entre 2000 e 2025 nas principais bases de dados biomédicas.

**Resultados:**

A sarcopenia na cirrose resulta de interação multifatorial envolvendo hiperamoniemia, inflamação sistêmica, resistência anabólica, disfunção hormonal, alterações mitocondriais, desnutrição e inatividade física. A tomografia computadorizada permanece o padrão-ouro para avaliação da massa muscular, embora métodos funcionais, como força de preensão manual e testes de desempenho físico, apresentem forte valor prognóstico. Abordagens multimodais são recomendadas para melhor acurácia diagnóstica. Intervenções nutricionais e exercício físico estruturado constituem a base do tratamento, enquanto terapias farmacológicas e biomarcadores emergentes, como miostatina, irisina e perfis metabolômicos, ainda carecem de validação clínica robusta.

**Conclusão:**

A sarcopenia é um determinante independente e potencialmente modificável do prognóstico na cirrose. A padronização dos critérios diagnósticos, a incorporação de avaliações multimodais e o desenvolvimento de intervenções terapêuticas direcionadas são essenciais para melhorar os desfechos clínicos.

## INTRODUCTION

Cirrhosis profoundly alters metabolic homeostasis, leading to a spectrum of nutritional and functional impairments in which sarcopenia has emerged as a central and independent determinant of prognosis. Far from being a secondary manifestation of advanced disease, sarcopenia contributes directly to increased mortality, higher rates of hepatic decompensation, infections, and inferior quality of life^1^ ^,^ ^2^. Its prevalence increases with disease severity and is particularly high among liver transplant candidates, in whom it predicts waitlist mortality and posttransplant complications more accurately than traditional scores such as the Model for End Stage Liver Disease (MELD)^3^ ^,^ ^4^.

Despite its clinical relevance, the field remains hindered by conceptual and methodological inconsistencies. Definitions vary across societies, cutoffs for muscle mass differ between populations, and functional assessments are inconsistently incorporated into diagnostic algorithms. The overlap between sarcopenia, malnutrition, and hepatic frailty further complicates clinical interpretation, as these entities share pathophysiological pathways but represent distinct constructs with different prognostic implications^5^ ^,^ ^6^.

Recent advances in metabolomics, artificial intelligence-enhanced imaging, and molecular biomarkers offer opportunities to refine diagnosis and personalize therapy. However, most remain investigational, with limited external validation and uncertain clinical applicability. Similarly, therapeutic strategies are constrained by small, heterogeneous trials, lack of standardized exercise protocols, and limited evidence supporting pharmacological interventions.

Given these gaps, a critical synthesis is needed to clarify current knowledge, highlight methodological limitations, and outline priorities for research and clinical practice. This review integrates mechanistic, diagnostic, and therapeutic perspectives, highlighting methodological limitations and outlining priorities for clinical practice and research.

## METHODS

This narrative review was conducted following methodological principles recommended for highquality evidence syntheses in hepatology. A literature search was performed in PubMed/MEDLINE, Scopus, and Web of Science from January 2000 to December 2025. Search terms included: “sarcopenia”, “cirrhosis”, “muscle mass”, “frailty”, “myostatin”, “irisin”, “metabolomics”, “liver transplantation”, “nutrition”, “exercise” and “hyperammonemia”. Boolean operators and MeSH terms were applied to maximize sensitivity.

Inclusion criteria were original studies (observational, interventional, translational), systematic reviews and meta-analyses, and clinical guidelines from major societies (AASLD, EASL, ESPEN, EWGSOP), involving adult patients with chronic liver disease or cirrhosis. Exclusion criteria were pediatric populations, non-peer-reviewed material, conference abstracts without full data, and studies lacking clear diagnostic criteria for sarcopenia.

Two reviewers independently screened titles and abstracts, with full text evaluation for eligible studies. Discrepancies were resolved by consensus. Given the narrative design, no formal risk-of-bias assessment was performed; however, methodological limitations of included studies were critically appraised and explicitly discussed. The review prioritizes mechanistic insights supported by experimental and clinical evidence, diagnostic tools with validated prognostic relevance, therapeutic interventions with reproducible outcomes, and emerging biomarkers with translational potential.

### Definition and prevalence

The conceptualization of sarcopenia has evolved substantially over the past three decades. Initially described by Rosenberg in 1989 as age-related loss of muscle mass, it is now recognized as a multidimensional condition encompassing reductions in muscle strength, quantity, and quality^5^ ^,^ ^7^. Contemporary definitions emphasize that muscle strength is a more robust predictor of adverse outcomes than muscle mass alone, a distinction particularly relevant in cirrhosis, where metabolic and inflammatory disturbances disproportionately impair contractile function.

In cirrhosis, sarcopenia represents the most clinically relevant nutritional and functional complication, with prevalence estimates ranging from 30% to 70% depending on diagnostic modality, population characteristics, and disease severity^8^. This wide variability reflects the absence of standardized cutoffs and the heterogeneity of assessment methods, which complicates comparisons across studies and limits implementation in routine care.

Sarcopenic obesity characterized by excess adiposity masking muscle depletion has gained prominence in the context of metabolic dysfunction associated steatotic liver disease (MASLD). This phenotype is frequently underdiagnosed and confers a prognosis comparable to or worse than isolated sarcopenia, with higher mortality and increased risk of hepatic decompensation^9^ ^,^ ^10^.

Frailty, although conceptually distinct, overlaps substantially with sarcopenia. The Liver Frailty Index (LFI) has demonstrated superior predictive accuracy for waitlist mortality compared with MELD, underscoring the importance of integrating functional assessment into risk stratification^4^ ^,^ ^6^. However, the boundaries between frailty, malnutrition, and sarcopenia remain blurred, and the lack of harmonized definitions continues to hinder clinical translation.

Overall, despite increasing recognition, the field remains limited by inconsistent diagnostic criteria, population-specific cutoffs, and methodological variability. Standardization is urgently needed to enable meaningful comparisons, guide therapeutic decisions, and support regulatory acceptance of sarcopenia as a modifiable prognostic determinant in cirrhosis.

### Pathophysiology

Sarcopenia in cirrhosis arises from a complex interplay of metabolic, inflammatory, hormonal, and functional disturbances. Although individual mechanisms have been well described, their relative contributions and interactions remain incompletely understood, reflecting the multifactorial nature of muscle wasting in chronic liver disease.

**Hyperammonemia and Mitochondrial Dysfunctions are central drivers of muscle catabolism**. Elevated ammonia levels impair mitochondrial function, reduce ATP production, and activate autophagy, leading to accelerated proteolysis. Ammonia also suppresses the mTOR pathway and increases myostatin expression, contributing to anabolic resistance^11^ ^,^ ^12^.

**Systemic Inflammation and Oxidative Stress,** characterized by elevated Tumor Necrosis Factor α (**TNF-**α), Interleukin 6 (**IL-6**), and endotoxemia, activates the ubiquitin-proteasome system and inhibits Insulin-Like Growth Factor-1 (**IGF-1**) signaling, further impairing protein synthesis. Chronic inflammation also contributes to mitochondrial dysfunction and oxidative stress, exacerbating muscle degradation^10^.

**Hormonal dysregulation** plays a substantial role. Cirrhotic patients frequently exhibit hypogonadism, insulin resistance, and reduced growth hormone/IGF-1 axis activity. These alterations impair muscle anabolism and promote fat infiltration into muscle fibers, reducing contractile quality^13^.

**Nutritional Deficits and Accelerated Starvation,** including reduced caloric intake, early satiety due to ascites, altered taste, and malabsorption, accelerate muscle breakdown. The cirrhotic liver quickly depletes glycogen stores, leading to early fasting and increased reliance on gluconeogenesis from muscle protein, a phenomenon known as “accelerated starvation”^14^.

**Physical inactivity**, often driven by fatigue, frailty, and hepatic encephalopathy, further contributes to muscle atrophy and functional decline. Reduced activity exacerbates anabolic resistance and impairs mitochondrial biogenesis.

Although these mechanisms are increasingly well characterized, significant gaps remain. Most mechanistic insights derive from animal models or small observational studies, and the relative contribution of each pathway likely varies across disease stages and phenotypes. Translational research linking molecular pathways to therapeutic targets remains limited, underscoring the need for mechanistically informed clinical trials.

**FIGURE 1:**
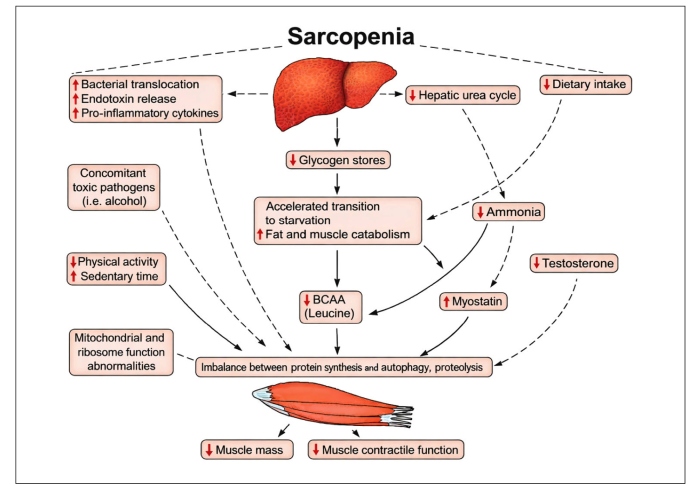
Integrated pathophysiological model of sarcopenia in cirrhosis. This figure illustrates the major biological pathways contributing to sarcopenia in cirrhosis, including hyperammonemia-induced mitochondrial dysfunction, systemic inflammation, anabolic resistance through impaired IGF-1 and mTOR signaling, hormonal disturbances, malnutrition, and reduced physical activity. The diagram highlights the interconnected nature of these mechanisms and their cumulative impact on muscle protein synthesis and degradation^55^. IGF-1: insulin-like growth factor-1. mTOR: mechanistic target of rapamycin. BCAA: branched-chain amino acid.

The Figure 1 illustrates a pathophysiological model of sarcopenia in cirrhosis.

### Diagnostic assessment

Accurate assessment of sarcopenia in cirrhosis is challenging due to fluid retention, body composition alterations, and the lack of universally accepted diagnostic criteria. Current methods evaluate structural and functional components, each with strengths and limitations. 

### Structural assessment

**Computed tomography (CT)** at the L3 vertebral level is considered the gold standard for quantifying skeletal muscle mass. The skeletal muscle index (SMI) derived from CT correlates strongly with mortality and waitlist outcomes^3^ ^,^ ^4^. SMI cutoffs (<50 cm^2^/m^2^ for men; <39 cm^2^/m^2^ for women) predict mortality in transplant candidates^3^. However, CT is limited by cost, radiation exposure, and the need for specialized software. Cutoffs remain population-specific, and their prognostic validity varies across cohorts.

**Dual-energy X-ray absorptiometry (DEXA)** provides whole-body composition estimates but is significantly affected by ascites and edema, reducing accuracy in advanced cirrhosis^4^.

Portable and radiation-free, ultrasound is promising but lacks standardized protocols and validated cutoffs. Recent studies suggest good reproducibility for quadriceps thickness, but external validation is still limited^15^.

**Bioelectrical Impedance Analysis (BIA)** is inexpensive and widely available but highly sensitive to hydration status, making it unreliable in patients with ascites or edema. Segmental multifrequency BIA improves accuracy but still lacks robust validation in cirrhosis^5^.

**Anthropometry:** anthropometric measures such as mid-arm muscle circumference (MAMC) and triceps skinfold thickness (TSF) are simple, low-cost, and unaffected by ascites^16^. Saueressig et al. proposed sex-specific MAMC cutoffs (<24.2 cm for men and <21.5 cm for women), which were independently associated with one-year mortality. These findings support the use of MAMC as a pragmatic tool in resource-limited settings, although external validation across diverse populations remains necessary. Recent multicenter Brazilian data demonstrated that MAMC predicts mortality independently of MELD-Na and Child-Pugh class, supporting its use in resource-limited settings^16^. However, interobserver variability and lack of universal cutoffs remain challenges.

In settings where imaging is unavailable, anthropometric measurements remain valuable. The EASL 2019 guidelines recommend mid-arm muscle circumference (MAMC), arm muscle area (AMA), and triceps skinfold thickness (TSF) as simple, low-cost methods unaffected by ascites or edema^17^. Among these, MAMC has shown superior prognostic value and good reproducibility.

### Functional assessment

Sarcopenia exerts a substantial impact on the natural history of cirrhosis, contributing to higher mortality, increased risk of decompensation, infections, and inferior post-transplant outcomes. Management requires a multidimensional approach combining nutritional optimization, structured exercise, and targeted treatment of hepatic complications. However, the evidence base remains limited by small sample sizes, heterogeneous methodologies, and lack of standardized endpoints. Functional measures are increasingly recognized as essential components of sarcopenia diagnosis.

**Handgrip strength** is simple, reproducible, and strongly associated with mortality, frailty, and hospitalization^13^ ^,^ ^18^. 

**Physical performance tests** including Gait Speed, the Short Physical Performance Battery, the Timed Up and Go test, and the Sixminute walk test-capture global functional capacity and predict clinical outcomes^5^ ^,^ ^19^. Data have shown that low daily step count and reduced performance on the sixminute walk test are associated with increased risk of hospitalization and mortality in community-dwelling patients with cirrhosis^6^.

**Dynapenia**, an isolated reduction in muscle strength, has been demonstrated as an independent predictor of adverse liver-related events, including ascites, encephalopathy, variceal bleeding, liver failure, and hepatocellular carcinoma, surpassing skeletal muscle mass as a functional predictor^15^ ^,^ ^20^.

While structural and functional assessments are essential for identifying sarcopenia, they represent only part of the broader nutritional and metabolic disturbances observed in cirrhosis. Because muscle loss frequently coexists with malnutrition and systemic inflammation, a comprehensive evaluation must also incorporate validated nutritional frameworks and direct measures of muscle mass. The following section summarizes current approaches to assessing malnutrition and muscle mass, highlighting their prognostic relevance and practical limitations.

Table 1 summarizes diagnostic methods for assessing sarcopenia in cirrhosis.

**TABLE 1:** Diagnostic methods for assessing sarcopenia in cirrhosis.This table summarizes the principal tools used to evaluate sarcopenia in patients with cirrhosis, including imaging-based, anthropometric, and functional assessments. For each method, the table details measurement type, commonly used cutoffs, major advantages and limitations, prognostic associations, and preferred clinical contexts. Cutoffs and prognostic data are derived from studies specifically conducted in cirrhotic populations.

Method	Measurement	Cutoffs	Major Advantages	Major Limitations	Prognostic	Clinical Context	Key Reference
CT (L3 Skeletal Muscle Index)	Muscle mass cross-sectional area at L3 normalized by height	Men <50 cm²/m²; women <39 cm²/m²	Gold standard; strong prognostic value; widely validated	Radiation; cost; requires software; not suitable for serial monitoring	Waitlist mortality; posttransplant mortality; complications	Liver transplant evaluation; patients undergoing CT for HCC surveillance	Carey EJ et al., Hepatology 2017
CT (Psoas or Paraspinal Muscle Area)	Segmental muscle mass	Cohortspecific percentiles	Uses existing imaging; feasible retrospectively	Lower reproducibility; less robust than total SMI	Mortality; infections; length of stay	Centers without full morphometry software	Bhanji RA et al., Hepatology 2019
DEXA	Total and appendicular lean mass	No cirrhosisspecific consensus	Low radiation; reproducible; whole-body composition	Strongly affected by ascites/edema; equipment required	Frailty; reduced physical function	Research settings; combined bone/muscle assessment	Lai JC et al., Hepatology 2017
Bioelectrical Impedance Analysis (BIA)	Estimated lean mass	Cohortspecific	Portable; inexpensive; useful for serial follow-up	Highly hydration-dependent; ascites overestimates lean mass	Moderate association with outcomes	Outpatient screening; lowresource settings	Merli M et al., Eur J Gastroenterol Hepatol 2015
MidArm Muscle Circumference (MAMC)	Peripheral muscle mass (anthropometry)	Men <24.2 cm; women <21.5 cm	Very low cost; unaffected by ascites; bedside applicability	Interobserver variability; regional cutoffs	One-year mortality; functional decline	Resourcelimited settings; routine screening	Saueressig MG et al., Clin Nutr 2024
Handgrip Strength (HGS)	Muscle strength	<27 kg (men); <16 kg (women)	Simple; inexpensive; strong predictor of mortality	Does not measure mass; requires cooperation	Mortality; hospitalization; frailty	Screening; pretransplant evaluation	Ha J et al., Liver Int 2018
Short Physical Performance Battery (SPPB)	Physical performance	<8 points	Integrates balance, gait, and strength; strong prognostic value	Requires space/time; less specific to muscle	Mortality; falls; hospitalization	Frailty assessment; transplant evaluation	Kirk A et al., J Hepatol 2024
Six-minute Walk Test (6MWT)	Functional capacity	<250-300 m	Reflects global functional reserve; responsive to interventions	Influenced by cardiopulmonary disease	Hospitalization; mortality	Exercise programs; pretransplant assessment	Lin S et al., J Hepatol 2022
Liver Frailty Index (LFI)	Composite frailty score	≥4.5-4.6	Validated; superior to MELD for waitlist mortality	Does not measure muscle mass	Waitlist mortality; hospitalization	Transplant candidate evaluation	Lai JC et al., Hepatology 2017
Ultrasound (Quadriceps Thickness)	Peripheral muscle mass	No validated cutoffs	No radiation; portable; repeatable	Operatordependent; limited validation	Correlates with CT and strength	Research; centers with expertise	Nishikawa H et al., J Gastroenterol Hepatol 2021

CT: computed tomography. SMI: skeletal muscle index. DEXA: dual-energy X-ray absorptiometry. BIA: bioelectrical impedance analysis. MAMC: mid-arm muscle circumference. HGS: handgrip strength. SPPB: short physical performance battery. 6MWT: six-minute walk test. LFI: liver frailty index. HCC: hepatocellular carcinoma.

### Nutritional assessment and muscle mass

 Leading international nutrition societies have proposed the **Global Leadership Initiative on Malnutrition (GLIM)**, which defines diagnostic based on the combination of phenotypic criteria (weight loss, low Body Mass Index, and reduced muscle mass) and etiologic criteria (reduced food intake or systemic inflammation). A diagnosis of malnutrition requires at least one criterion from each category, with muscle mass assessment preferably performed using imaging methods^21^.

### Artificial intelligence and automated imaging

Advances in artificial intelligence and deep learning models have enabled automated analysis of CT images, identifying and quantifying muscle mass with high precision and reproducibility. Onishi et al. (2025) demonstrated that models such as DeepLabv3 and EfficientNetV2-XL accurately segmented skeletal muscle area on CT scans and estimated skeletal muscle index with high sensitivity and specificity^22^. These technologies may standardize sarcopenia assessment, reduce observer bias, and facilitate integration into clinical practice as prognostic and monitoring tools.

Despite methodological advances, no single method fully satisfies the criteria of accuracy, feasibility, and broad applicability in cirrhosis^16^. CT-based morphometry remains the most validated tool but is impractical for routine screening. DEXA is substantially affected by ascites and fluid overload, whereas BIA depends heavily on hydration status. Anthropometric measures such as MAMC offer low-cost alternatives but lack universal cutoffs and are susceptible to interobserver variability. Thus, a multimodal approach-integrating structural measures, functional assessments, and nutritional evaluation offer the most comprehensive and cost-effective strategy for detecting sarcopenia in cirrhosis.

Although current diagnostic tools provide valuable structural, functional, and nutritional information, they remain limited by variability in cutoffs, dependence on operator expertise, and inconsistent applicability across clinical settings. These limitations have stimulated growing interest in molecular and metabolic biomarkers capable of capturing early or subclinical muscle alterations.

### Emerging biomarkers in cirrhosis-associated sarcopenia

The search for reliable biomarkers of sarcopenia in cirrhosis has intensified in recent years, driven by the limitations of imaging-based assessment and the need for tools capable of capturing early metabolic alterations. Among the most promising candidates are myostatin, irisin, and metabolomic signatures, each reflecting distinct aspects of muscle biology. However, despite encouraging preliminary data, none has yet achieved sufficient validation for routine clinical use.

**Myostatin (GDF-8),** a Transforming Growth Factor β (**TGF-**β) family member, is a potent inhibitor of muscle growth. In cirrhosis, circulating and intramuscular myostatin levels are elevated, likely driven by chronic inflammation, hyperammonemia, and insulin resistance^23^. Increased myostatin expression suppresses Akt/mTOR signaling, exacerbating anabolic resistance, and accelerating muscle loss. Clinical studies demonstrate that higher serum myostatin correlates with lower muscle mass, reduced strength, and increased mortality^20^. However, heterogeneity in assays, lack of standardized thresholds, and confounding by renal dysfunction limit its current applicability. Therapeutic inhibition of myostatin remains experimental, with early-phase trials in non-cirrhotic populations showing mixed results.

**Irisin,** a myokine released during physical activity, promotes mitochondrial biogenesis and improves insulin sensitivity. Cirrhotic patients exhibit significantly reduced irisin levels, which correlate with lower lean mass, impaired functional performance, and systemic inflammation^24^ ^,^ ^25^. Experimental models suggest that irisin enhances Akt/mTOR signaling and downregulates myostatin, positioning it as a potential mediator of exercise-induced benefits.

However, clinical data remain limited, and methodological inconsistencies in irisin quantification hinder cross-study comparisons. Whether irisin can serve as a therapeutic target or biomarker of response to rehabilitation requires further investigation.

**Metabolomic Signatures** offers a comprehensive view of metabolic derangements in cirrhosis-associated sarcopenia. Typical profiles include reduced branched-chain amino acids (BCAAs), increased aromatic amino acids, and alterations in lipid and energy metabolism^26^. These patterns reflect mitochondrial dysfunction, impaired protein synthesis, and altered substrate utilization.

Recent studies integrating metabolomics with machine-learning approaches have identified panels of metabolites capable of discriminating sarcopenic from non-sarcopenic cirrhotic patients with high accuracy, particularly involving alterations in amino-acid-derived metabolites and lipid species, including ceramide-related pathways^26^. Nevertheless, small sample sizes, lack of external validation, and variability in analytical platforms limit clinical translation.

Metabolomics therefore enables an integrative and non-invasive assessment of muscular and hepatic metabolic status, representing a new frontier in the diagnostic and prognostic evaluation of cirrhosis-associated sarcopenia. However, the absence of analytical standardization and validated cutoffs currently limits its integration into clinical practice. Myostatin, irisin, and metabolomic profiles represent a new generation of biomarkers that expand our understanding of the pathophysiology of sarcopenia in cirrhosis by linking metabolic, inflammatory, and hormonal pathways.

Although emerging biomarkers provide valuable mechanistic insights, their clinical utility remains constrained by methodological heterogeneity, absence of validated cutoffs, and limited reproducibility. At present, they should be viewed as complementary research tools rather than diagnostic standards. Large, multicenter studies integrating biomarkers with imaging and functional assessments are needed to advance precision phenotyping and guide targeted interventions.

### Clinical and therapeutic implications

Sarcopenia exerts a profound impact on clinical outcomes in cirrhosis, including increased mortality, higher rates of hepatic decompensation, infections, and inferior post-transplant survival^2^. The management of sarcopenia in patients with cirrhosis is multidimensional, involving supervised physical exercise, nutritional interventions, and pharmacological therapies. Despite its prognostic relevance, therapeutic strategies remain limited by low-quality evidence, heterogeneous study designs, and lack of standardized protocols^27^.

### Nutritional interventions

**Caloric and Protein Targets:** nutritional optimization is a cornerstone of management. Current guidelines recommend caloric intake ≥35 kcal/kg/day and protein intake ≥1.2-1.5 g/kg/day^28^ ^,^ ^29^. Frequent meals and late-evening snacks mitigate accelerated starvation and reduce muscle proteolysis^14^. Plant-based proteins may reduce ammonia production and improve HE control, although evidence remains modest (Iqbal et al., 2021).

**Prolonged fasting** should be avoided in patients with cirrhosis, as hepatocellular dysfunction impairs hepatic glycogen synthesis, storage, and mobilization. As a result, the cirrhotic liver has markedly reduced glycogen reserves, which are depleted within hours of fasting. Consequently, the body enters an early catabolic state, relying on muscle protein breakdown for gluconeogenesis. This phenomenon occurs even after short fasting periods (6-10 hours) and is known as “pseudo-fasting”, characterized by a rapid metabolic shift toward muscle consumption^28^ Enhanced proteolysis directly contributes to the progression of sarcopenia, worsening loss of lean body mass, physical function, and clinical outcomes. Therefore, frequent meals every 3-4 hours and inclusion of a late-evening snack are recommended to reduce time in a catabolic state, preserve muscle mass, and support improved nutritional and metabolic outcomes^14^.

**Dietary protein** is a major source of intestinal ammonia production, a key factor in the occurrence and/or control of hepatic encephalopathy (HE). Recommended protein intake may come from plant-based or animal sources. Compared to animal protein, plant protein contains higher levels of arginine, which in turn increases urea synthesis. Furthermore, it has a higher fiber content, which consequently creates an acidic colonic environment that favors ammonia excretion. Finally, plant protein also contains lower levels of methionine and tryptophan. Collectively, these effects lead to lower circulating levels of ammonia and mercaptans, both involved in mediating HE^30^.

**Branchedchain amino acids (BCAAs)**: particularly leucine-activate mTOR signaling and stimulate muscle protein synthesis. Meta-analyses show modest improvements in hepatic encephalopathy and quality of life but inconsistent effects on muscle mass or survival^31^. Their role appears more supportive than transformative. BCAAs are among the most anabolic essential amino acids, particularly leucine, which plays a central role in skeletal muscle protein synthesis^32^. Beyond serving as protein substrates, BCAAs act as pharmacological nutrients and promote albumin synthesis via mTOR activation, improve albumin redox status and function in cirrhotic patients, stimulate hepatocyte growth factor secretion by hepatic stellate cells, modulate immune function, and may attenuate insulin resistance and hepatocarcinogenesis in cirrhosis. Some data also suggest that BCAAs support liver regeneration after injury. Despite their pleiotropic actions, these benefits have not consistently translated into improvements in hard clinical outcomes^33^. Accordingly, their use should be individualized, with BCAAs serving primarily as supportive therapy in patients with hepatic encephalopathy or insufficient protein intake^34^.

**Deficiencies in vitamins** and trace elements are highly prevalent in cirrhosis and contribute to neuromuscular dysfunction, regardless of disease etiology, and particularly prevalent in advanced disease, cholestasis, or acute illness^35^.

**Thiamine (vitamin B1)** is a water-soluble vitamin involved in nerve propagation and serves as a key cofactor in amino acid and carbohydrate metabolism. Vitamin B1 deficiency is especially common in alcohol-related cirrhosis due to insufficient intake and alcohol-induced damage of gastrointestinal thiamine absorption^36^. Thiamine deficiency leads to peripheral neuropathy, cardiomyopathy, or Wernicke encephalopathy^36^. **Vitamin A** plays essential roles in multiple metabolic pathways, from vision to gene transcription. The liver is the primary storage site of vitamin A, particularly within hepatic stellate cells^37^. **Vitamin D** deficiency correlates with increased risk of infections, bone disease (osteoporosis and osteopenia), and mortality^33^ ^,^ ^38^.

**Magnesium deficiency** is associated with muscle cramps, a frequent complaint among cirrhotic patients. Manganese is excreted via bile and can accumulate in the brain, particularly the basal ganglia, causing encephalopathy and extrapyramidal symptoms mimicking Parkinsonism^28^. **Zinc deficiency**, frequent in advanced cirrhosis, is linked to impaired immune function, altered ammonia metabolism, and worse transplant-free survival. These abnormalities warrant systematic screening and targeted supplementation^39^.

### Exercise and physical rehabilitation: evidence-based perspective

Exercise has emerged as the most consistently effective and biologically coherent intervention for sarcopenia in cirrhosis. Unlike pharmacological therapies that target isolated pathways, structured physical activity exerts broad, multi-system effects that directly counteract the metabolic, inflammatory, hormonal, and mitochondrial disturbances characteristic of chronic liver disease. Over the past decade, a growing body of clinical and translational research has reframed exercise not as an optional adjunct, but as a central therapeutic pillar in the management of cirrhosis-assciated muscle loss.

The rationale for exercise in cirrhosis is grounded in robust mechanistic data. Resistance training activates the mechanistic target of rapamycin (mTOR) pathway, stimulating muscle protein synthesis and promoting satellite cell proliferation-processes that are markedly suppressed in cirrhosis due to hyperammonemia, inflammation, and hormonal dysregulation^11^ ^,^ ^12^. Aerobic exercise complements these effects by enhancing mitochondrial biogenesis through Peroxisome Proliferator-Activated Receptor Gamma Coactivator-1 Alpha (**PGC-1α**) activation, improving oxidative phosphorylation, and reducing ammonia-induced mitochondrial dysfunction^20^. Exercise also attenuates systemic inflammation, lowering circulating TNF-α and IL-6, and improves insulin sensitivity, thereby reducing proteasomemediated muscle catabolism^10^.

Although randomized trials remain small, their findings are remarkably consistent. In a landmark study, Berzigotti et al. (2016)^40^ ^,^ ^41^ demonstrated that a 12-week supervised exercise program improved aerobic capacity, muscle strength, and quality of life in patients with compensated cirrhosis, without increasing portal pressure. Similar benefits were observed in trials by Zenith et al. (2014)^42^ and Román et al*.* (2016)^43^, which reported significant gains in sixminute walk distance, peak VO_2_, and handgrip strength.

Prehabilitation-structured exercise combined with nutritional and educational interventions-has gained relevance in transplant hepatology. Data has demonstrated that prehabilitation improves the Liver Frailty Index, reduces waitlist hospitalizations, and enhances posttransplant recovery^44^. Even modest increases in daily physical activity, such as achieving >5,000 steps/day, are associated with lower mortality and fewer complications^6^. These findings underscore the prognostic significance of functional reserve and highlight exercise as a modifiable determinant of transplant outcomes.

Given the logistical barriers faced by many patients-including fatigue, frailty, and lack of access to specialized centers-home-based and tele-rehabilitation programs have emerged as practical alternatives. Studies using wearable accelerometers, remote coaching, and elastic-band resistance training have demonstrated high adherence and clinically meaningful improvements in strength and mobility^45^. These models expand access while maintaining safety and efficacy.

Although standardized protocols are lacking, evidence supports a multimodal approach combining resistance, aerobic, and functional training:

- **Resistance training:** 2-3 sessions/week, 6-10 exercises targeting major muscle groups, 2-3 sets of 8-12 repetitions^40^.
- **Aerobic training:** 20-40 minutes/session at moderate intensity (40-60% VO_2_max), such as walking or cycling^42^.
- **Functional training:** balance, gait, and mobility exercises to reduce frailty and fall risk^44^.

Exercise should be avoided during acute decompensation, and intensity should be adjusted based on fatigue, hemodynamic tolerance, and encephalopathy status.

Despite strong mechanistic rationale and consistent functional benefits, several limitations persist. Trials are small, heterogeneous, and often short in duration. Long-term effects on survival, decompensation, and transplant-free mortality remain uncertain. Standardized endpoints-such as changes in muscle strength, physical performance, and frailty indices-are needed to harmonize future research. Integration of biomarkers, imaging, and wearable technology may enable more precise monitoring of treatment response.

Among all available interventions, exercise stands out as the most effective, safe, and broadly applicable therapy for sarcopenia in cirrhosis. It addresses the multifactorial drivers of muscle loss, improves functional capacity, enhances quality of life, and strengthens transplant readiness. While pharmacological therapies remain adjunctive, exercise combined with nutritional optimization forms the foundation of evidence-based management.

### Pharmacological therapies: promise and limitations

Pharmacological therapies for sarcopenia in cirrhosis remain an area of active investigation, driven by the multifactorial nature of muscle wasting and the limited efficacy of nutritional and exercise interventions alone. Although several agents demonstrate biological plausibility and encouraging preclinical results, translation into meaningful clinical benefit has been inconsistent. To date, no pharmacological therapy is approved specifically for cirrhosis-associated sarcopenia, and most interventions remain adjunctive rather than disease-modifying.

**Testosterone and androgen-based therapies:** Hypogonadism is common in men with cirrhosis and contributes to reduced muscle mass, bone density, and functional capacity. Testosterone replacement increases lean body mass and bone mineral density in small, randomized trials, but improvements in muscle strength or clinical outcomes are inconsistent^46^ ^,^ ^47^. Potential benefits include stimulation of protein synthesis via androgen receptor activation, increased IGF-1 signaling, improved erythropoiesis and energy levels.

However, safety concerns limit widespread use. Testosterone may exacerbate fluid retention, worsen portal hypertension, and theoretically stimulate hepatocellular carcinoma growth. Transdermal formulations may offer more stable pharmacokinetics, but evidence remains insufficient. Therapy should be restricted to carefully selected hypogonadal men with close monitoring.

**Selective Androgen receptor modulators (SARMs)** provide tissue-selective anabolic effects with fewer androgenic side effects. Agents such as enobosarm increase lean mass in older adults and chronic disease populations, but gains in strength and physical performance have been modest^48^. Key limitations include hepatotoxicity signals in early trials, lack of data in cirrhosis and uncertain long-term safety. Given altered drug metabolism in cirrhosis, SARMs remain experimental and should not be used outside clinical trials.

**Myostatin and ActRII inhibitors:** myostatin, a negative regulator of muscle growth, is upregulated in cirrhosis and contributes to anabolic resistance. Inhibiting myostatin or its receptor (ActRII) increases muscle mass in non-cirrhotic populations, but functional improvements have been inconsistent across clinical trials^49^. Challenges include edema and fluid retention observed in some programs, potential offtarget effects on cardiac and smooth muscle and absence of studies in cirrhosis, where safety concerns are amplified. Despite strong mechanistic rationale, these agents remain investigational.

**Growth hormone and IGF-1 Axis modulation.** Growth hormone (GH) resistance and reduced IGF-1 levels are hallmarks of cirrhosis. GH supplementation increases IGF-1 and lean mass in small studies but is limited by high cost, risk of insulin resistance and potential for worsening portal hypertension. IGF-1 therapy is theoretically attractive but limited by short halflife and concerns about tumorigenesis^10^ ^,^ ^50^. Neither approach is recommended in clinical practice.

**Ammonia-lowering agents.** Hyperammonemia contributes directly to muscle catabolism by impairing mitochondrial function, increasing autophagy, and upregulating myostatin. Lactulose and rifaximin improve hepatic encephalopathy but have not consistently demonstrated improvements in muscle mass or strength^51^ ^,^ ^52^. L-ornithine L-aspartate (LOLA) enhances ammonia detoxification and increases muscle protein synthesis in animal models, but human data remain limited and heterogeneous. Overall, ammonia-lowering therapies may support muscle preservation indirectly but are not sufficient as stand-alone treatments for sarcopenia. Rifaximin has emerging observational evidence suggesting association with improved muscle mass in cirrhosis with HE, but prospective trials are still needed^51^ ^,^ ^52^.

**Metabolic and mitochondrial modulators have** been evaluated as potential therapeutic adjuncts in cirrhosis. L-carnitine may ameliorate fatigue and enhance ammonia metabolism, although evidence for improvements in muscle mass or function is minimal^53^. Antioxidants, including vitamin E and N-acetylcysteine, provide theoretical mechanistic benefit but lack convincing clinical data^10^ ^,^ ^50^. Metformin improves insulin sensitivity and may reduce hepatocellular carcinoma risk, yet no studies demonstrate efficacy in sarcopenia^10^. These agents should therefore be considered investigational and are not recommended for the targeted treatment of sarcopenia.

**Anti-inflammatory and anti-catabolic** strategies have been explored considering the contribution of systemic inflammation to muscle wasting. TNF-α inhibitors and IL-6 antagonists exhibit anabolic effects in other chronic conditions, yet their applicability in cirrhosis is constrained by a high risk of infection. Similarly, autophagy inhibitors and proteasome modulators demonstrate encouraging preclinical activity but remain unsupported by human studies^4^ ^,^ ^49^ ^,^ ^50^.

### Why Have pharmacological therapies not achieved consistent clinical benefit?

Multiple factors contribute to the modest performance of pharmacological therapies in sarcopenia, including its multifactorial pathophysiology, altered drug pharmacokinetics in cirrhosis, risks of fluid retention, infection, and hepatotoxicity, the lack of validated trial endpoints, heterogeneous patient cohorts, and the generally short duration of available studies. As a result, these agents currently serve as adjuncts rather than core therapeutic strategies.

**FIGURE 2:**
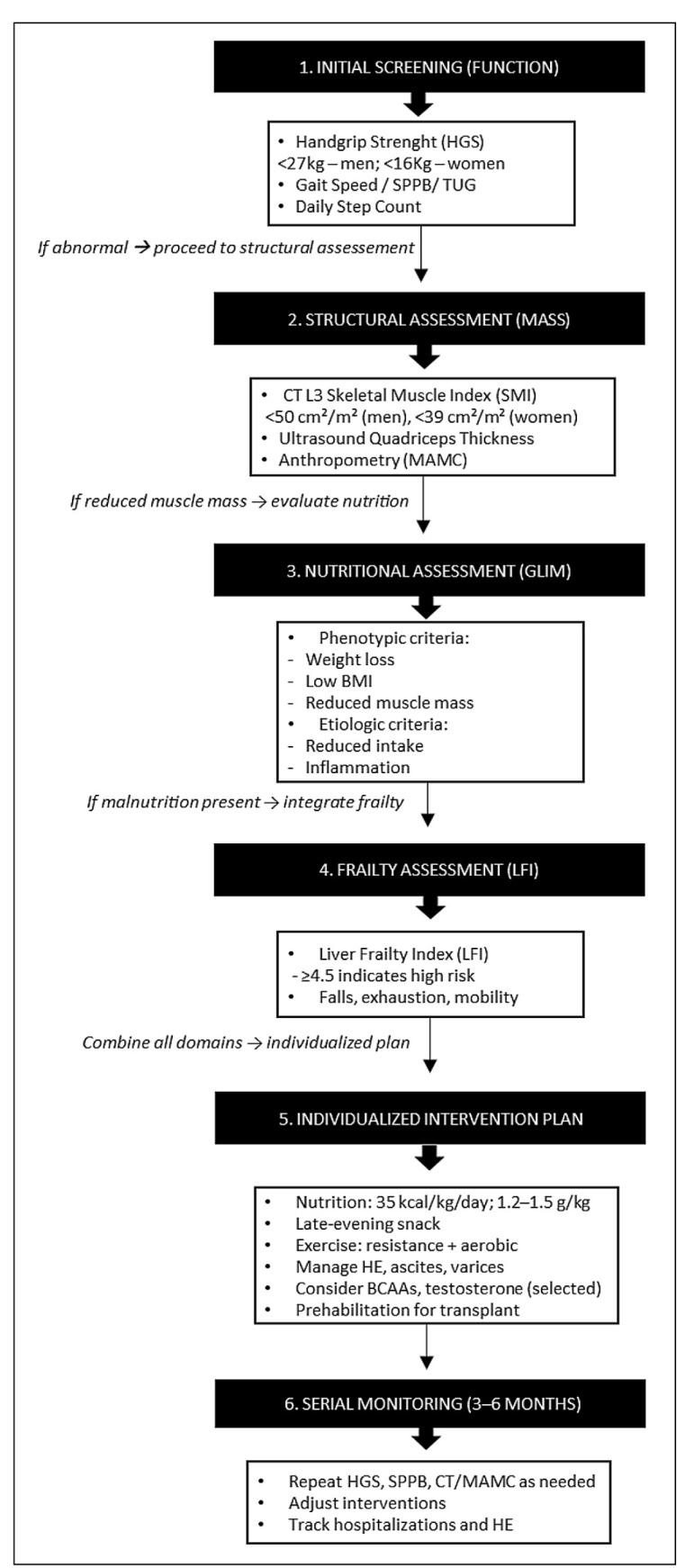
Proposed multimodal framework for the assessment and management of sarcopenia in cirrhosis.

**Current role in clinical practice.** Exercise and nutrition remain the cornerstone of management. Pharmacological interventions may be appropriate only for carefully selected subgroups-such as hypogonadal men or patients with hepatic encephalopathy receiving BCAAs-and most agents should be confined to clinical trial settings. Advancing the field will require mechanistically grounded trials, harmonized diagnostic criteria, and composite endpoints capable of capturing meaningful clinical change. Table 2 summarizes available data, and Figure 2 outlines a proposed multimodal management framework.

**TABLE 2:** Therapeutic interventions for sarcopenia in cirrhosis: evidence, clinical effects, and limitations.

Intervention	Intervention	Population	Study Design	Outcomes	Main Findings	Key Limitations	Key Reference
Supervised Resistance Training	Exercise (strength + aerobic)	Compensated cirrhosis (Child A/B)	Small randomized trials (8-16 weeks)	Muscle mass, strength, VO^2^, quality of life	↑ strength, ↑ lean mass, ↑ aerobic capacity; safe	Small samples; heterogeneous protocols; limited data in Child C	Berzigotti A et al., J Hepatol 2017
Multimodal Prehabilitation (Exercise + Nutrition)	Integrated rehabilitation	Pretransplant candidates	Prospective cohorts	Frailty, LFI, hospitalization, transplant readiness	Improved physical performance; possible reduction in admissions	No large RCTs; heterogeneous interventions	Aamann L et al., Liver Int 2020
HighProtein Diet (1.2-1.5 g/kg/day)	Nutritional optimization	Compensated and decompensated cirrhosis	Clinical trials and cohorts	Nitrogen balance, lean mass, HE	Improved nitrogen balance; no worsening of HE	Variable adherence; heterogeneous diets	ESPEN Guidelines 2020; Bunchorntavakul C, Clin Liver Dis 2020
LateEvening Snack	Meal timing intervention	Cirrhotics at nutritional risk	Small clinical trials	Substrate oxidation, nitrogen balance	Reduced nocturnal catabolism; improved protein balance	Small studies; surrogate outcomes	Tsien C et al., Hepatology 2012
Oral BranchedChain Amino Acids (BCAAs)	Amino acid supplementation	Cirrhotics with HE or sarcopenia	RCTs; Cochrane metaanalysis	HE, quality of life, muscle mass, survival	Modest improvement in HE/QoL; inconsistent muscle effects	Heterogeneous formulations; adherence issues	Gluud LL et al., Cochrane 2017
PlantBased Protein Diet	Protein source modulation	Cirrhotics with HE	Small crossover trials	HE, ammonia, protein tolerance	Better tolerance; possible ammonia reduction	Small samples; limited muscle outcomes	Iqbal U et al., Nutr Rev 2021
Testosterone Replacement (Hypogonadal Men)	Hormonal therapy	Men with cirrhosis and hypogonadism	Small RCTs	Lean mass, strength, bone density	↑ lean mass and bone density	Safety concerns (fluid retention, HCC risk)	Sinclair M et al., Clin Endocrinol 2016
AmmoniaLowering Agents (Lactulose, Rifaximin, LOLA)	Metabolic modulation	Cirrhotics with HE; animal models for muscle	RCTs for HE; preclinical for muscle	HE, ammonia, muscle mass (animals)	Improved HE; rifaximin+LOLA↑ muscle mass in animals	No human trials with muscle endpoints	Kumar A et al., Hepatology 2017
Myostatin Inhibitors / ActRII Antibodies	Experimental anabolic therapy	Noncirrhotic chronic disease populations	Phase II/III trials	Muscle mass, function	↑ muscle mass; inconsistent functional gains	No cirrhosis data; safety concerns	Bhanji RA et al., Hepatology 2019
Selective Androgen Receptor Modulators (SARMs)	Experimental anabolic therapy	Older adults, COPD, cancer	Phase II trials	Lean mass, strength	↑ lean mass; inconsistent strength effects	No cirrhosis data; hepatotoxicity risk	General sarcopenia reviews (no cirrhosis trials)
Structured PreTransplant Prehabilitation	Multimodal intervention	Liver transplant candidates	Cohort studies	LFI, hospital stay, posttransplant recovery	Improved LFI; possible shorter hospitalization	No RCTs; heterogeneous programs	Tandon P et al., J Hepatol 2021

This table provides an overview of available and emerging therapeutic strategies for sarcopenia in cirrhosis, including nutritional interventions, structured exercise programs, metabolic modulation, hormonal therapy, and experimental anabolic agents. For each intervention, the table outlines the population studied, study design, primary outcomes, main findings, key limitations, and approximate level of evidence. Evidence levels reflect the strength and consistency of published data in cirrhotic cohorts.

A stepwise clinical algorithm integrating functional screening, structural assessment of muscle mass, nutritional evaluation using GLIM criteria, frailty assessment with the Liver Frailty Index, and individualized therapeutic interventions. The framework emphasizes early detection, multidisciplinary management, and serial monitoring to optimize outcomes in patients with cirrhosis.

CT: computed tomography. SMI: skeletal muscle index. MAMC: mid-arm muscle circumference. GLIM: Global Leadership Initiative on Malnutrition. LFI: Liver Frailty Index. HE: hepatic encephalopathy. BCAA: branched-chain amino acids.

### Integrating sarcopenia into prognostic models

Although survival is the most robust and universally accepted outcome, it is relatively insensitive to interventions aimed at preserving or improving muscle mass. Patient-centered outcomes-such as quality of life, functional capacity, physical performance, and hospitalization rates-may better capture clinically meaningful benefits and should be incorporated into future trial designs.

A challenge is the integration of sarcopenia into established prognostic models for cirrhosis. Traditional scores such as MELD do not account for muscle mass, strength, or functional reserve, despite strong evidence that sarcopenia independently predicts mortality and waitlist dropout. Composite models such as “MELD-Sarcopenia” have been proposed, but they lack external validation and are limited by the absence of standardized diagnostic criteria and universally accepted cutoffs^54^.

Functional indices including the Liver Frailty Index consistently outperform MELD in predicting waitlist mortality, underscoring the prognostic value of incorporating muscle-related variables into risk stratification^4^. Integrating structural, functional, and metabolic markers of sarcopenia into predictive models may refine prognostication, improve transplant prioritization, and support more individualized clinical decision-making.

## CONCLUSION

Sarcopenia is a major, yet underrecognized, determinant of morbidity and mortality in cirrhosis. Its pathophysiology reflects a complex interplay of hyperammonemia, systemic inflammation, hormonal dysregulation, mitochondrial dysfunction, malnutrition, and physical inactivity. Although CT-based morphometry remains the gold standard for assessing muscle mass, no single diagnostic tool captures the full spectrum of structural, functional, and metabolic alterations. A multimodal approach integrating imaging, functional performance, and nutritional assessment is therefore essential.

Therapeutic strategies remain limited by low-quality evidence, small heterogeneous trials, and lack of standardized protocols. Nutritional optimization and structured exercise programs consistently improve strength, functional capacity, and quality of life, and should be considered first-line therapy. Pharmacological interventions-including testosterone, BCAAs, myostatin inhibitors, and SARMs-show biological plausibility but lack robust clinical validation. Emerging biomarkers and artificial intelligence-based imaging tools offer exciting opportunities for precision phenotyping but require further study.

Future research must prioritize standardized diagnostic criteria, mechanistically informed clinical trials, and integrated interventions that address the multifactorial nature of sarcopenia. Recognizing sarcopenia as a modifiable prognostic factor and incorporating it into clinical decision-making-particularly in transplant evaluation-represent critical steps toward improving outcomes in patients with cirrhosis.

A focused research agenda is essential to advance the field. Priority areas include standardizing diagnostic criteria and population-specific cutoffs, validating AI-based imaging tools across diverse cohorts, and defining clinically meaningful endpoints for interventional trials. Developing structured and reproducible exercise protocols, evaluating multimodal strategies that combine nutrition, exercise, and pharmacotherapy, and validating biomarkers for diagnosis and monitoring are also critical. Finally, incorporating sarcopenia into transplant prioritization models may improve risk stratification and support more individualized clinical decision-making.

## Data Availability

not applicable - the study did not use research data
